# Reduced FGF9 Leads to Kidney Injury Through Regulating Renal Tubular Epithelial Cell EMT in Diabetes

**DOI:** 10.1111/jcmm.70856

**Published:** 2025-09-22

**Authors:** Wen‐qing Chen, Chengyang Sun, Xiaotan Zhang, Xunjia Ye, Yuzhen Liu, Hui‐di Wang, Lichao Liu, Danrui Li, Jingyun Wang, Meiting Shi, Fang Yang, Christoph Reichetzeder, Berthold Hocher, Xuesong Yang, Baozhang Guan, Guang Wang

**Affiliations:** ^1^ The First Affiliated Hospital of Jinan University, School of Medicine Jinan University Guangzhou China; ^2^ Linyi Central Hospital Linyi China; ^3^ International Joint Laboratory for Embryonic Development and Prenatal Medicine, Division of Histology and Embryology, School of Medicine Jinan University Guangzhou China; ^4^ Key Laboratory for Regenerative Medicine of the Ministry of Education Jinan University Guangzhou China; ^5^ Fifth Department of Medicine (Nephrology/Endocrinology/Rheumatology, Pneumology), University Medical Center Mannheim University of Heidelberg Mannheim Germany; ^6^ Institute for Clinical Research and Systems Medicine HMU—Health and Medical University Potsdam Germany; ^7^ Reproductive, Genetic Hospital of CITIC‐Xiangya Changsha China; ^8^ Institute of Medical Diagnostics, IMD Berlin Germany; ^9^ School of Basic Medical Science Central South University Changsha China; ^10^ Guangdong‐Hong Kong Metabolism and Reproduction Joint Laboratory, Guangdong Second Provincial General Hospital, School of Medicine Jinan University Guangzhou China

**Keywords:** diabetic nephropathy, epithelial‐to‐mesenchymal transition, fibroblast growth factor 9, renal fibrosis

## Abstract

Diabetic nephropathy (DN) stands out as one of the most prevalent and severe chronic microvascular complications associated with diabetes, serving as the primary cause of end‐stage renal disease (ESRD) in developed and developing countries. However, the precise pathogenesis remains incompletely elucidated. Our study suggests FGF9 as a key gene in DN using bioinformatics analysis. We found a negative correlation between FGF9 and serum creatinine and a positive one with glomerular filtration rate in DN patients. FGF9 expression was lower in DN patients' glomeruli and tubules. High FGFR expression in renal tubular cells, along with increased α‐SMA and TGF‐β1, indicates a role for the epithelial‐to‐mesenchymal transition (EMT) process in diabetes mellitus (DM) mouse renal tubular epithelial cells. Subsequently, we modulated FGF9 in HK2 cells under different glucose conditions. The genes regulated by FGF9 were identified (LOX, HIF1α, THBS1, TGFβ2 and ITGβ1) through RNA sequencing analysis. It was suggested that FGF9 promotes the development of renal EMT probably through regulating these genes. Overall, FGF9 could be a biomarker and therapeutic target for DN.

## 1 Introduction

The global prevalence of diabetes in individuals aged 20–79 years was estimated to be 10.5% in 2021, affecting approximately 536.6 million people, with projections suggesting an increase to 12.2% (783.2 million) by 2045 [1]. Diabetic nephropathy (DN) stands as one of the most prevalent and severe chronic microvascular complications linked to diabetes and it remains the leading cause of end‐stage renal disease (ESRD) worldwide [2]. It is estimated that approximately 20%–50% of individuals with diabetes may eventually develop DN [3]. Furthermore, nearly 50% of DN patients progress to ESRD, significantly elevating the mortality risk among individuals with DN [4].

DN affects both glomeruli and tubules, with common pathological changes encompassing glomerulosclerosis and tubulointerstitial fibrosis (TIF), eventually progressing to renal sclerosis [5, 6]. TIF is characterised by the deposition of extracellular matrix (ECM) [7, 8]. A growing body of evidence supports the involvement of epithelial‐to‐mesenchymal transition (EMT) in tubular epithelial cells (TECs) in the progression of DN. During EMT, TECs undergo a transformation where they begin to express fibroblast markers and lose their epithelial characteristics [9]. This transformation ultimately leads to ECM remodelling and the development of TIF in DN. Complex interactions involving numerous growth factors, cytokines, hormones, extracellular signalling molecules and transcriptional regulators have been extensively documented in the context of EMT and fibrosis [10, 11]. Among the various factors identified, the profibrotic cytokine TGF‐β1, either alone or in conjunction with other mediators in tubular epithelial cells, serves as the primary driver responsible for inducing EMT [12, 13]. However, the molecular mechanisms underlying TIF in DN remain unclear [14].

The fibroblast growth factor (FGF) family constitutes a group of structurally similar and multifunctional signalling molecules that regulate numerous developmental, physiological and pathological processes in humans. Most of these molecules signal through the activation of four transmembrane tyrosine kinase receptors, designated FGFR1–FGFR4 [15]. While some associations of FGFs in the context of DN are known, the exact role and mechanisms of FGFs in DN remain unclear [16, 17]. Although the FGF9 subfamily is known to be critical for EMT [18] and kidney development [19], whether it plays a role in DN is still unknown. In this study, we combined mRNA sequence analysis and hub gene screening, utilising human tissue samples, a diabetic kidney injury mouse model and HK2 cell lines, to investigate the role of FGF9 in driving renal tubular epithelial EMT in DN.

## 2 Materials and Methods

### 2.1 Microarray Data Preprocessing

The microarray data was obtained from the Gene Expression Omnibus (GEO) database and includes the following datasets: GSE30528, GSE1009 and GSE47183 [20]. The GSE1009 dataset consists of samples obtained from autopsy, specifically three pairs of normally functioning renal and diabetic renal samples. The GSE30528 dataset comprises 9 renal tissue samples from DN patients and 13 renal tissue samples from control humans. On the other hand, GSE47183 consists of 7 renal tissue samples from DN patients and 14 renal tissue samples from patients who underwent tumour nephrectomy. Differential expression analysis was conducted using the limma package in R software. To identify statistically significant differentially expressed genes (DEGs), the criteria used were |logFC| ≥ 1.5 and a *p* value < 0.01. We used DAVID (https://david.ncifcrf.gov/) [21] to envision GO and KEGG pathway function enrichment analyses of DEGs.

### 2.2 Protein–Protein Interaction (PPI) Network Analysis and Hub Gene Screening

The overlapping differentially expressed genes (DEGs) from GSE1009 and GSE30528 were subjected to a protein–protein interaction (PPI) network analysis using the STRING database (https://www.string‐db.org/). To identify hub genes within the PPI network, the cytoHubba plugin of Cytoscape (version 3.9.0) was utilised. The top 15 hub genes from each of these algorithms were selected. These hub genes were then screened using the ‘UpSetR’ package to identify the common hub genes shared among the different algorithms [22]. Afterwards, the DEGs were classified into hub genes using the overlapping genes of the LASSO algorithm with the glmnet package. Subsequently, the prediction of hub genes was further validated in dataset GSE47183.

### 2.3 Human Tissue Collection

Human kidney specimens were obtained from kidney biopsies conducted at the First Affiliated Hospital of Jinan University. The specimens included normal kidney tissue from donors who had tragically passed away due to car accidents (normal group) and damaged kidney regions from patients diagnosed with diabetes (DN group, stage 5 kidney disease). Serum samples were collected from two distinct groups: healthy individuals and individuals with DN (stage 5 kidney disease). These individuals were hospitalised in the Department of Nephrology and for health check‐ups at the Physical Examination Center of the First Affiliated Hospital of Jinan University, China, during the period spanning from June 1, 2023, to October 1, 2023. The inclusion and exclusion criteria for tissue collection are listed in Table 1. This study was approved by the Ethics Committee of Overseas Hospital, Jinan University, China (approval number: KY‐2023‐222) and conducted in accordance with the Declaration of Helsinki.

**TABLE 1:** Inclusion and exclusion criteria.

Inclusion criteria Age: 20–65 years Meet diagnostic criteria for diabetes Patients with microalbuminuria and macroalbuminuria meet the diagnostic criteria for DN Obtain patient informed consent
Exclusion criteria Confirmed primary kidney disease Other systemic disorders that can lead to proteinuria Combined with cardiovascular and cerebrovascular, liver, kidney and other serious primary diseases, blood system diseases and tumours

### 2.4 Enzyme‐Linked Immunosorbent Assay (ELISA)

Two millilitre of participants' early morning fasting peripheral venous blood was collected in an anticoagulation tube, and the supernatant was taken at 1300 rpm for 15 min in a 1.5 mL EP tube and stored in a −80°C refrigerator. The FGF9 is quantified in the serum using UV spectrophotometry with a detection kit following the manufacturer's instructions (XY9H0133, XYBiotechnology, China).

### 2.5 Experimental Animal

C57BL/6J male mice utilised in this study were sourced from the Institute of Laboratory Animal Science at Jinan University in Guangzhou, China. These mice were maintained in an animal facility with controlled conditions, including a stable room temperature, humidity level (50%–60%), a 12:12 h light–dark cycle and were provided with standard food pellets and water. Eight‐week‐old mice (*n* = 7) were chosen for the induction of diabetes mellitus (DM). This was achieved by administering STZ (Sigma‐Aldrich, St. Louis, MO, USA), which was dissolved in 0.01 mol/L citrate buffer with a pH of 4.5, at a dose of 75 mg/kg body weight for three consecutive days. Blood glucose levels were monitored using the Roche Accu‐Chek Aviva Blood Glucose System (Roche, Penzberg, BY, Germany) for a duration of 7 days following STZ injection. Mice with blood glucose levels exceeding 16 mM were classified as type 1 diabetic and were raised to 16 weeks for subsequent experiments [23]. A control group comprised of normal mice that were fed normally and injected with the same volume of 0.01 mol/L citrate buffer (*n* = 10). All experimental mice were finally euthanised by cervical dislocation. The housing conditions for all mice were consistent with the previously mentioned standards [24, 25, 26, 27]. Mouse serum was collected by cardiac puncture before the mice were killed under anaesthesia. Metabolic cages were used to collect 24‐h urine from mice. Blood samples were centrifuged for 10 min at 1000 rpm and blood serum creatinine was determined using an automatic biochemistry analyser (Roche Diagnostic Systems, cobas8000 c702, Japan). Urine microalbumin concentration was measured using a kit from the Jiancheng Institute of Biotechnology (EO38‐1‐1, Nanjing, China) following standard protocols.

All research involving the use of animals in this study was performed by the procedures of the Ethical Committee for Animal Experimentation, Jinan University.

### 2.6 Histology

16‐week‐old mouse kidneys, representing both control and DM groups, were fixed in 4% paraformaldehyde at 4°C for 24 h. Subsequently, the specimens underwent a series of processing steps, including dehydration, clearing in xylene and embedding in paraffin wax. The paraffin‐embedded specimens were then sliced into 5 μm sections using a rotary microtome (Leica, Germany). These sections were subjected to various staining techniques, including haematoxylin and eosin (H&E) staining, periodic acid Schiff (PAS) reaction staining, Masson's trichrome dye staining (Masson staining) and immunohistochemical staining (Table S2). To determine the extent of collagen deposition and fibrosis in renal tissue, we established a mesangial matrix index. This index was statistically determined from measurements of previously reported kidney sections stained with periodic acid‐Schiff dyes [28] and the relative area of areas stained positively by Masson. The relative area of positive staining was analysed on at least five randomly selected images (each image magnified 40 times) from three independent samples per group. Quantification of the staining was carried out using Image J and Image Pro Plus software, providing a reliable method for assessing the histological characteristics and alterations in the kidney tissue sections.

### 2.7 Cell Culture and Gene Transfection

HK2 cells (Human renal tubular epithelial cells) were procured from the Chinese Academy of Sciences Cell Bank (Shanghai, China). These HK2 cells were cultured in DMEM/F‐12 (Dulbecco's Modified Eagle Medium/Nutrient Mixture F‐12) from Gibco (Gaithersburg, MD, USA), supplemented with 5.5 mM glucose, 10% fetal bovine serum obtained from ExCell Bio, 100 U/mL of penicillin and 100 μg/mL of streptomycin. The cells were maintained in a humidified incubator at 37°C with 5% CO_2_ and cultured in six‐well plates at a concentration of 1 × 10^6^ cells/mL. They were subjected to 50 mM glucose (designated as the High Glucose group, HG) treatment [26]. For gene transfection, the HK2 cells were transfected using a control vector (NG) or FGF9‐overexpressing lentivirus vector (HG + OE‐FGF9) and si‐FGF9 (5′‐CUAUGUUGCAUUAAAUAAATT‐3′) with the assistance of lipofectamine 3000 (Invitrogen, Carlsbad, CA, USA) [26, 29]. The transfection procedure was conducted following the manufacturer's instructions.

### 2.8 RNA Sequencing Analysis

Whole‐genome gene expression analysis was conducted in HK2 cells from four groups: NG, si‐FGF9, HG and HG + OE‐FGF9. Total RNA was extracted using TRIzol and cDNA samples were sequenced using a HiSeq3000 sequencing system (Illumina). Transcriptome sequencing experiments were performed by Meiji Biomedical Technology Co. Ltd. (Shanghai, China). RNA sequencing (RNA‐seq) data were deposited in the National Center for Biotechnology Information GenBank (BioProject ID: GSE265918).

### 2.9 Transwell Migration/Invasion Assay

1 × 10^5^ HK2 cells, including control cells or transfected cells, were suspended in 100 μL of serum‐free medium. The choice of either the control or HG microenvironment was made for the cells in the inserts. The subsequent steps of the protocol were carried out in accordance with a previously published report [30].

### 2.10 Wound Healing Assay

5 × 10^5^ HK2 cells, including control and transfected cells, were seeded in 6‐well plates with either a control or high‐glucose (HG) microenvironment until they formed a confluent monolayer. Scratches were created in the monolayer using a 200 μL pipette tip, and cell migration was recorded at 0 and 48 h. Each treatment group was analysed in at least three wells, and images were captured using a Nikon Eclipse Ti‐U inverted microscope (Nikon Eclipse Ti‐U, Japan).

### 2.11 RNA Isolation and RT‐qPCR Analysis

Total RNA was extracted from human kidney tissue, mouse kidney tissue and HK2 cells using a Trizol kit from Invitrogen (USA), following the manufacturer's instructions. Subsequently, first‐strand cDNA was synthesised, resulting in a final volume of 20 μL, using an iScriptTM cDNA Synthesis Kit from BIO‐RAD (USA). Following reverse transcription, PCR amplification of the cDNA was conducted, following previously established protocols [31, 32]. SYBR Green quantitative PCR (qPCR) assays were performed using a PrimeScriptTM RT reagent kit from Takara (Japan). The specific primer sequences used in these assays were detailed in Table S1. The reverse transcription and amplification reactions were carried out using Bio‐Rad S1000TM thermal cycler equipment (Bio‐Rad, USA) and ABI 7000 thermal cyclers, respectively. To normalise the qRT‐PCR data, 18S ribosomal RNA was used as an internal reference. RT‐qPCR experiments were representative of findings from at least three independent experiments.

### 2.12 Western Blot Analysis

Total proteins were extracted from kidney tissues or HK2 cells using CytoBuster protein extraction reagent from Millipore (USA). The amount of total protein was measured using the BCA Protein Assay Kit (Thermo). For each Western blot analysis, equal amounts of total protein from each group were separated by 8%–12% SDS‐PAGE (sodium dodecyl sulphate‐polyacrylamide gel electrophoresis) and subsequently transferred electrically onto a PVDF membrane. The membrane was blocked with TBST (Tris‐buffered saline with 0.05% Tween 20, pH 7.4) containing 5% skim milk for 1 h at room temperature. Subsequently, the membrane was incubated with primary antibodies overnight at 4°C. The primary antibodies used were as follows: FGF9 (1:1000, Santa Cruz, sc‐398730, CA), α‐SMA (1:1000, Abcam, ab5694, UK), TGFβ1 (1:1000, Proteintech, 21898–1‐AP, USA), E‐cadherin (1:1000, DSHB, USA) and β‐actin (1:10000, Proteintech, USA). After three washes with TBST, the membrane was incubated with an appropriate secondary antibody (1:3000) for 2 h at room temperature. The detailed information on antibodies can be found in Table S3. Both primary and secondary antibodies were diluted with TBST. The blots were visualised under a Tennant Gel Imaging System using an ECL kit (WBKLS0500, Millipore), and the intensity of the bands was analysed using ImageJ software. WB results presented in the study were representative of findings from at least three independent experiments.

### 2.13 Data Analysis

In all experiments, we followed rigorous practices, conducting each experiment at least in triplicate for robust and reliable results. We employed a blinded outcome assessment to reduce potential bias in data interpretation. Coefficients of variation (CVs) were calculated for triplicate values in each experiment, and a grand mean CV was determined. We used SPSS 23.0 for statistical analyses and GraphPad Prism 9 for creating charts. Statistical significance was determined using paired *T*‐test or one‐way analysis of variance (ANOVA) followed by the Least‐Significance‐Difference test. Data presentation included mean ± SD or median with the interquartile range. Pearson's chi‐square tests compared rates between control and experimental data, with a significance level of *p* < 0.05 indicating statistical significance [33, 34, 35]. All animal experiment statistics data and the exact value of n are presented in Data S1.

## 3 Results

### 3.1 FGF9 Emerged as a Prominent Hub Gene in DN

**FIGURE 1:**
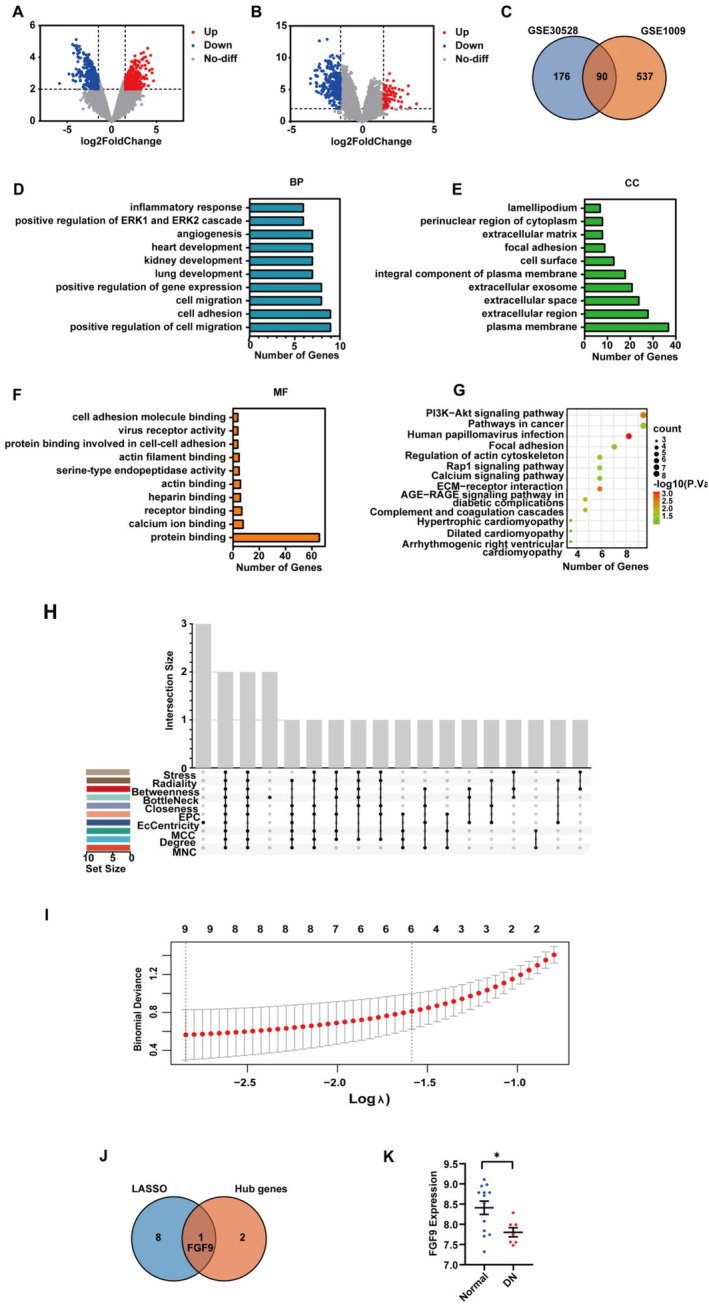
DEGs screening and bioinformatics analysis. (A, B): A volcano plot (significance vs. fold change) showing the DEGs (|logFc| ≥ 1.5 and *p* value < 0.01) in kidney between the controls and DN patients in GSE30528 and GSE1009 datasheets. (C) Identification of common DEGs in GSE30528 and GSE1009 datasheets. (D–F) Classifications of identified DEGs based on GO functional annotation. (G) Functional enrichment analyses of KEGG pathways. (H) Protein–Protein Interaction Network Analysis and hub Gene Screening based on Cytoscape and UpSetR software. (I) Candidate diagnostic biomarkers identified using the LASSO logistic regression algorithm. (J) Venn diagram showing the overlaps in the candidate diagnostic biomarkers identified using the LASSO logistic regression algorithm and ten algorithms of cytoscape. (K) FGF9 mRNA level verified in kidney between the controls and DN patients in GSE47183 datasheet. BP, Biological Process; CC, Cellular Component; CON, Control; DN, Diabetic nephropathy; MF, Molecular Function. *p < 0.05. Illustration in J was plotted by https://www.bioinformatics.com.cn (accessed November 10, 2023), an online platform for data analysis and visualisation. Illustration in C was created with TBtools and H was created with https://sangerbox.com.

To identify the differentially expressed genes (DEGs) between DN and normal human kidney tissues, we conducted an analysis of two independent RNA‐seq datasets (GSE30528 and GSE1009). The results revealed 266 differential genes in GSE30528 and 627 differential genes in GSE1009 (Figure 1A,B), with 90 DEGs common to both datasets (Figure 1C). To gain insights into the biological processes and pathways associated with these key DN genes, we performed functional enrichment analysis. The top 10 enriched terms in GO functional enrichment included positive regulation of cell migration, cell adhesion/migration, plasma membrane, extracellular region/space and protein binding across different processes (Figure 1D–F). Additionally, KEGG pathway analysis implicated the PI3K‐Akt signalling pathway, pathways in cancer, ECM‐receptor interaction, AGE‐RAGE signalling pathway and complement and coagulation cascades in DN pathophysiology (Figure 1G).

We conducted a comprehensive analysis of the common DEGs using the STRING online database (with a score threshold > 0.4) and Cytoscape software (Figure 1H). We employed the cytoHubba plugin within Cytoscape, applying 10 randomly selected algorithms (MCC, MNC, EPC, Degree, BottleNeck, Closeness, EcCentricity, Radiality, Betweenness and Stress) to score each node gene. The top 15 hub genes were identified using each algorithm, and ultimately, we pinpointed three hub genes (WT1, VEGFA and FGF9) using the ‘UpSetR’ package (Figure 1H).

In order to discover genetic features, a total of 90 genes in DEGs were included in the model constructed by the LASSO regression algorithm; only FGF9 is also a hub gene screened by Cytoscape (Figure 1I,J–L). Therefore, FGF9 has been identified as a potential diagnostic marker for DN. Subsequently, we also confirmed FGF9 expression utilising the GSE47183 dataset (Figure 1K) (Table S4).

### 3.2 FGF9 Expression Is Reduced in the Serum and Kidney Tissues of Patients With DN

To investigate the potential correlation between decreased FGF9 and DN, we examined the relationship between FGF9 expression and DN in a cohort of 51 healthy individuals and 49 DN patients, assessing their clinical and laboratory characteristics (Table 2). Through ELISA, we observed significantly lower serum FGF9 levels in DN patients compared to normal individuals. Furthermore, FGF9 exhibited a substantial decrease in kidney biopsy tissue from DN patients. Correlation analysis presented in Table 3 revealed that serum FGF9 expression exhibited a negative correlation with serum creatinine (*r* = −0.303, *p* < 0.001) levels, while it was positively correlated with estimated glomerular filtration rate (eGFR) (*r* = 0.243, *p* = 0.006). Immunohistochemical staining indicated that FGF9 expression was decreased both in renal glomeruli and tubules in the DN patients (Figure S1; Table S5). These collective findings suggest that FGF9 expressed in the kidney likely plays a crucial role in the development of DN, and low FGF9 expression may indeed serve as a risk factor for the progression of DN.

**TABLE 2:** Clinical and laboratory characteristics of the study population.

Variables	Total (*n* = 128)	Normal (*n* = 64)	DN (*n* = 64)	*p*
Sex, *n* (%)				< 0.001***
Female	80 (62)	53 (83)	27 (42)	
Male	48 (38)	11 (17)	37 (58)	
Age	46 (30.75, 59)	30.5 (24, 42)	58.5 (50, 64)	< 0.001***
GLU	5.12 (4.67, 7.9)	4.68 (4.5, 4.88)	7.9 (7, 9)	< 0.001***
ALT	15 (10, 23.25)	12 (10, 19.25)	19 (11, 26.25)	0.003**
AST	16 (14, 20)	15 (14, 18.25)	19 (14, 25)	0.005**
ALB	42.65 (36.6, 45.95)	45.75 (44.08, 47.8)	36.6 (33.7, 40.12)	< 0.001***
TG	0.98 (0.72, 1.52)	0.77 (0.66, 1)	1.46 (0.96, 1.9)	< 0.001***
TC	4.5 (3.69, 5.24)	4.66 (4.2, 5.13)	3.97 (3.26, 5.33)	0.032*
HDL	1.32 (0.99, 1.57)	1.5 (1.38, 1.72)	0.99 (0.84, 1.25)	< 0.001***
LDL	2.41 ± 0.78	2.58 ± 0.5	2.23 ± 0.96	0.014*
URIC	330.7 (275.75, 391.3)	291.5 (269.5, 336.25)	381.15 (313, 442.8)	< 0.001***
BUN	5.95 (4.5, 17.82)	4.6 (3.58, 5.2)	17.91 (8.72, 23.69)	< 0.001***
CREA	73.4 (47.63, 627)	47.55 (42.38, 54.28)	627 (333.68, 866.08)	< 0.001***
eGFR	99.19 (11.45, 125.38)	125.44 (119.44, 134)	11.2 (5.58, 47.95)	< 0.001***
FGF9	240.01 (193.46, 295.74)	263.98 (209.68, 310.79)	235.18 (191.41, 271.4)	0.026*

*Note:* Differences between the groups were compared with the Mann–Whitney *U* test or chi‐square test or Fisher's exact test.

Abbreviations: ALB, albumin; ALT, alanine aminotransferase; AST, aspartate aminotransferase; BUN, blood urea nitrogen; CERA, creatinine; DN, Diabetic nephropathy; eGFR, estimated glomerular filtration rate; FGF9, fibroblast growth factor 9; GLU, glucose; HDL‐c, high‐density lipoprotein cholesterol; LDL‐c, low‐density lipoprotein cholesterol; TC, serum total cholesterol; TG, triglyceride; UA, uric acid.

* *p* < 0.05.

** *p* < 0.01.

*** *p* < 0.001.

**TABLE 3:** The significant correlations between FGF‐9 and clinical indices in DN patients.

Variables	FGF9	
	*r*	*p*
GLU	−0.209	0.018*
ALT	−0.104	0.244
AST	−0.028	0.756
ALB	0.255	0.004**
TG	−0.244	0.005**
TC	0.078	0.38
HDL	0.133	0.135
LDL	0.115	0.196
URIC	−0.173	0.05
BUN	−0.155	0.081
CREA	−0.303	< 0.001***
eGFR	0.243	0.006**

*Note:* Partial correlation analysis was performed between GLU, ALT, AST, ALB, TG, TC, HDL‐c, LDL‐c, UA, eGFR, BUN, CREA and FGF9.

* *p* < 0.05.

** *p* < 0.01.

*** *p* < 0.001.

### 3.3 The FGF9‐FGFR Signalling Pathway Was Suppressed in a Mouse Model of Diabetic Kidney Injury

**FIGURE 2:**
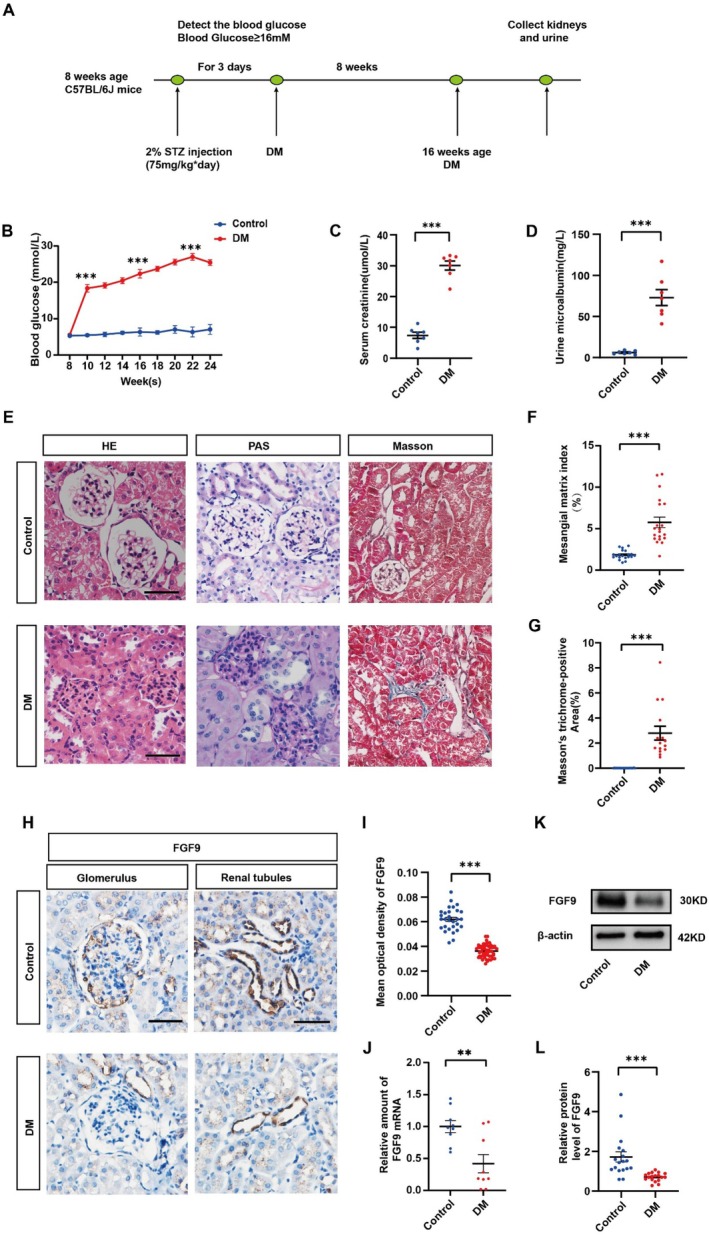
Microstructures, renal function and FGF9 expression in kidney of Control and DM mouse model. (A) Schematic illustration of the establishment of STZ‐induced DM mouse model. (B) Graph showing blood glucose levels at week 8–24 weeks following STZ administration among C57BL/6J mice. (C, D) The bar charts showing the Serum creatinine (C) and Urine microalbumin (D) among Control and DM mice. (E) Representative micrographs of H&E, PAS and Masson stained renal glomeruli and tubules from Control and DM mice. (F–G) The bar charts showing the Bowman's space (F) and Masson positive area (G) among Control and DM mice. (H) FGF9 immunohistochemical staining on the cross‐sections of kidney from Control and DM mice. The panels show FGF9 expression in renal glomerulus and tubule respectively. (I) Bar chart showing the comparison of mean optical density values of FGF9 immunohistochemical staining between Control and DM mice. (J–L) RT‐qPCR and Western blot data showing the mRNA and protein expression levels of FGF9 in kidney of Control and DM mice. Scale bar = 50 μm in C; 50 μm in F. *p < 0.05, **p < 0.01, ***p < 0.01.

To further research the mechanism between FGF9 and the development of DN, we examined the expression levels of FGF9 and FGFRs in a diabetes mouse model (DM group) induced by STZ injection (Figure 2A). Subsequently, we assessed the rise in blood glucose and changes in renal function, and observed morphological changes in glomeruli and tubules, demonstrating that hyperglycaemia leads to significant ultrastructural alterations in the kidneys (Figure 2B–E) (Table S6). The serum creatinine (Figure 2C) and urine microalbumin (Figure 2D) of diabetic mice were increased. Diabetic mice displayed augmented mesangial matrix, as indicated by PAS staining (Figure 2F). Masson staining revealed an increased collagen area in renal interstitial tissue of diabetic mice compared to normal control mice (Figure 2G). Collectively, these findings confirm the successful establishment of a DM mouse model. Furthermore, we assessed FGF9 expression through immunohistochemistry, RT‐qPCR and Western blot, all of which consistently showed a significant decrease in FGF9 levels in the kidneys of diabetic mice (Figure 2H–L). These results further support the establishment of a diabetic kidney injury mouse model, aligning with FGF9 expression levels in DN patients.

**FIGURE 3:**
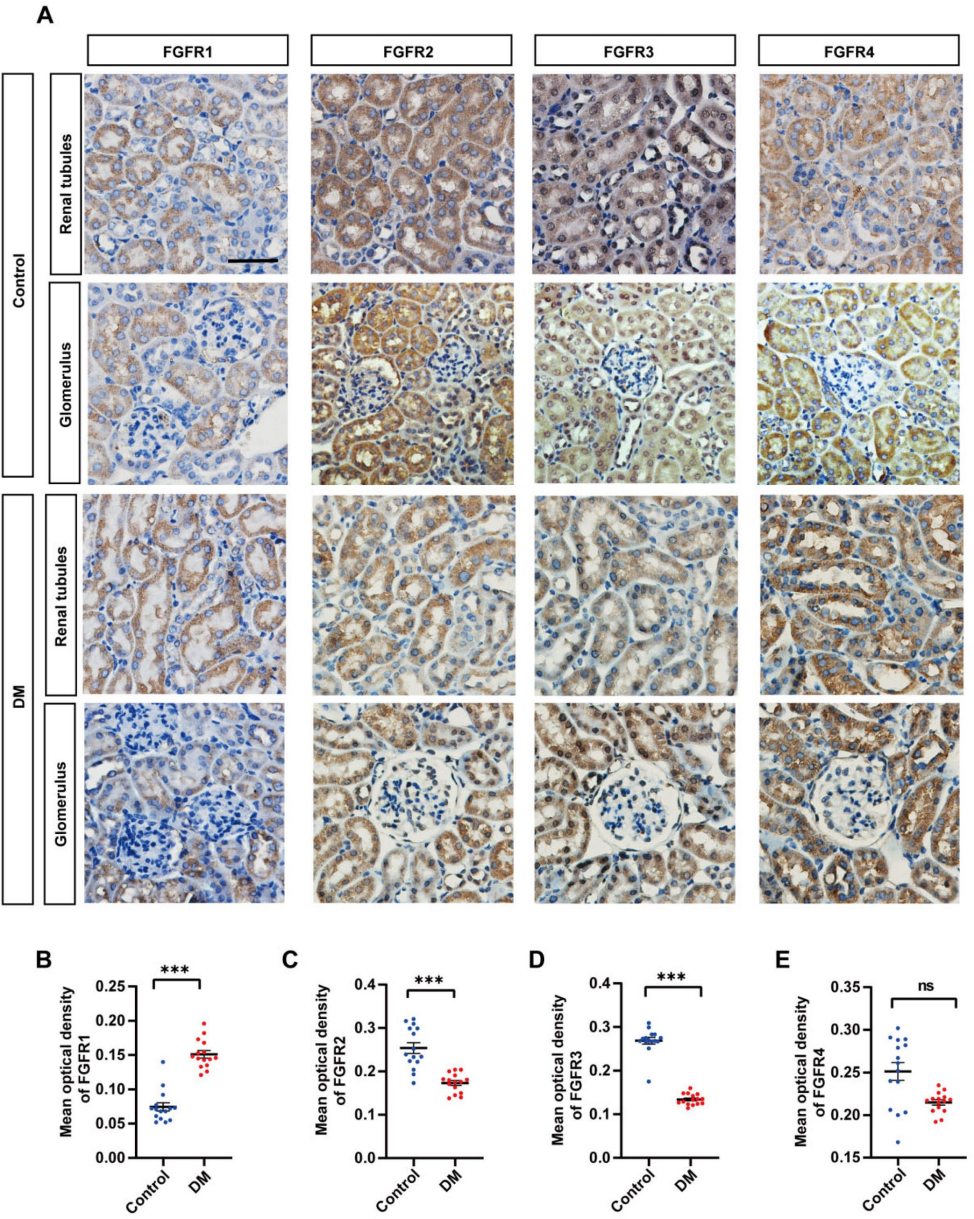
FGF receptors expression in renal tubule of Control and DM mouse model. (A) FGFR1, FGFR2, FGFR3 and FGFR4 immunohistochemical staining on the cross‐sections of kidney from control and DM mice. (B–E) The bar chart showing the comparison of mean optical density values of FGFR1‐4 immunohistochemical staining between renal tubule of Control and DM mice. Scale bar = 50 μm in A. ***p < 0.01; ns = no significance.

Additionally, we examined the expression of FGFRs in the diabetic kidney injury mouse model. FGFR1‐4 were primarily expressed in tubular epithelial cells rather than in the glomeruli of diabetic kidney injury mice (Figure 3A–E) (Table S7). Immunohistochemical analysis revealed an upregulation in the expression of FGFR1, while the expression of FGFR2 and FGFR3 exhibited a decrease, which closely paralleled the observed FGF9 expression pattern.

### 3.4 FGF9 Was Involved in the EMT of Renal Tubular Epithelial Cells

**FIGURE 4:**
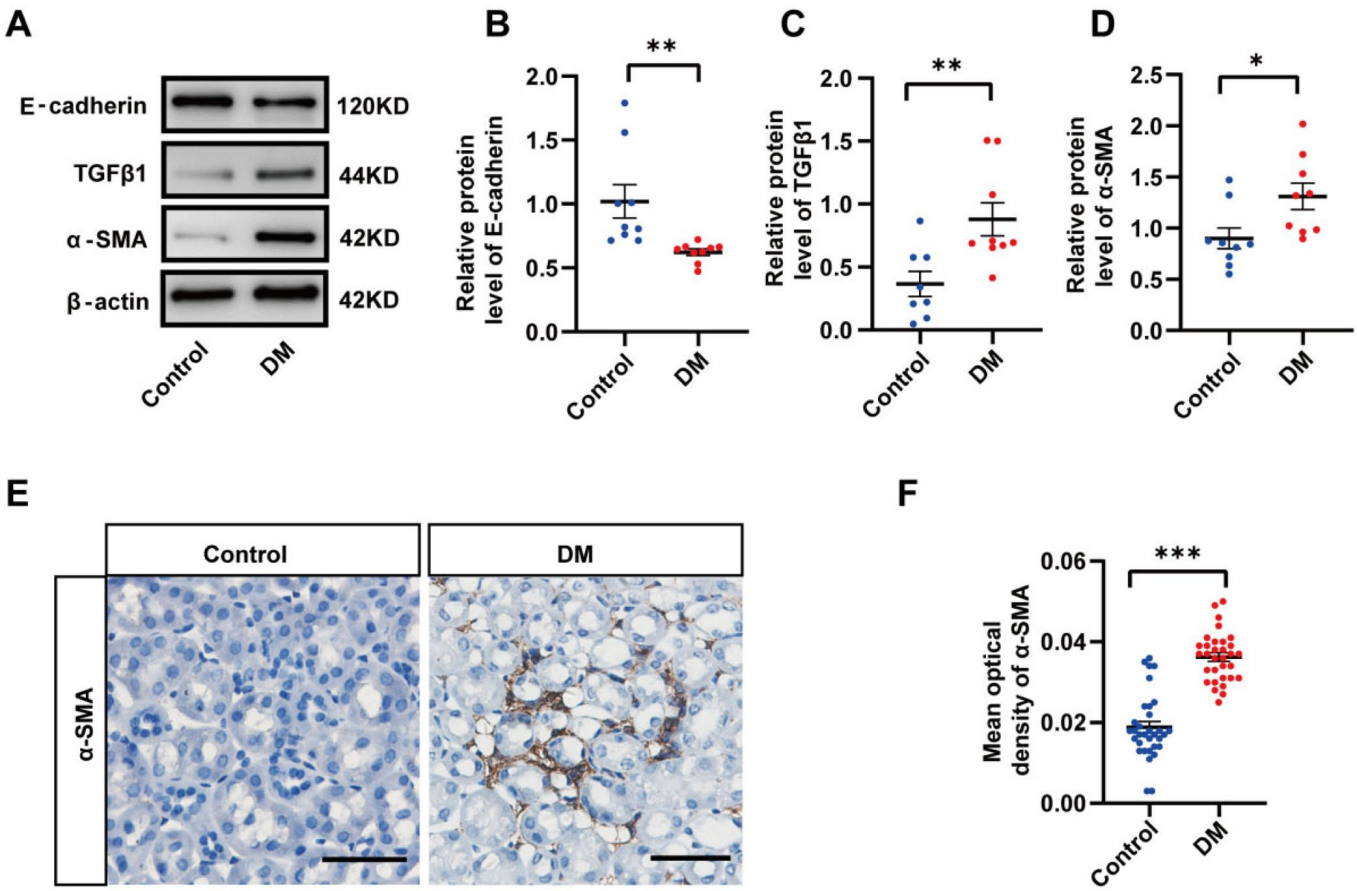
Renal interstitial fibrosis and profibrotic‐associated proteins in Control and DM mouse model. (A) Western blot showing the E‐cadherin, TGF‐β1 and ɑ‐SMA protein expression levels in kidney of Control and DM mice. (B) Immunofluorescent staining for ɑ‐ SMA from Control and DM mice. (F) Bar charts showing ɑ‐SMA mean optical density values between renal tubule of Control and DM mice. (A–D) Scale bars = 50 μm in A. *p < 0.05, **p < 0.01, ***p < 0.001.

Renal tubular cells are often the primary targets of kidney injury, with some undergoing the EMT process and transforming into activated myofibroblasts. This transformation constitutes a crucial mechanism driving the progression of renal fibrosis [36, 37, 38]. Thus, we investigated the EMT process in renal tubular epithelial cells. Western blot results indicated an increase in α‐SMA and TGF‐β1 levels, while E‐cadherin exhibited a decrease in kidneys of diabetic mice (Figure 4A–D). Immunohistochemical analysis revealed an upregulation of α‐SMA in the diabetic kidney injury model (Figure 4E,F) (Table S8). These findings collectively suggest the occurrence of fibrosis and EMT in the kidney.

**FIGURE 5:**
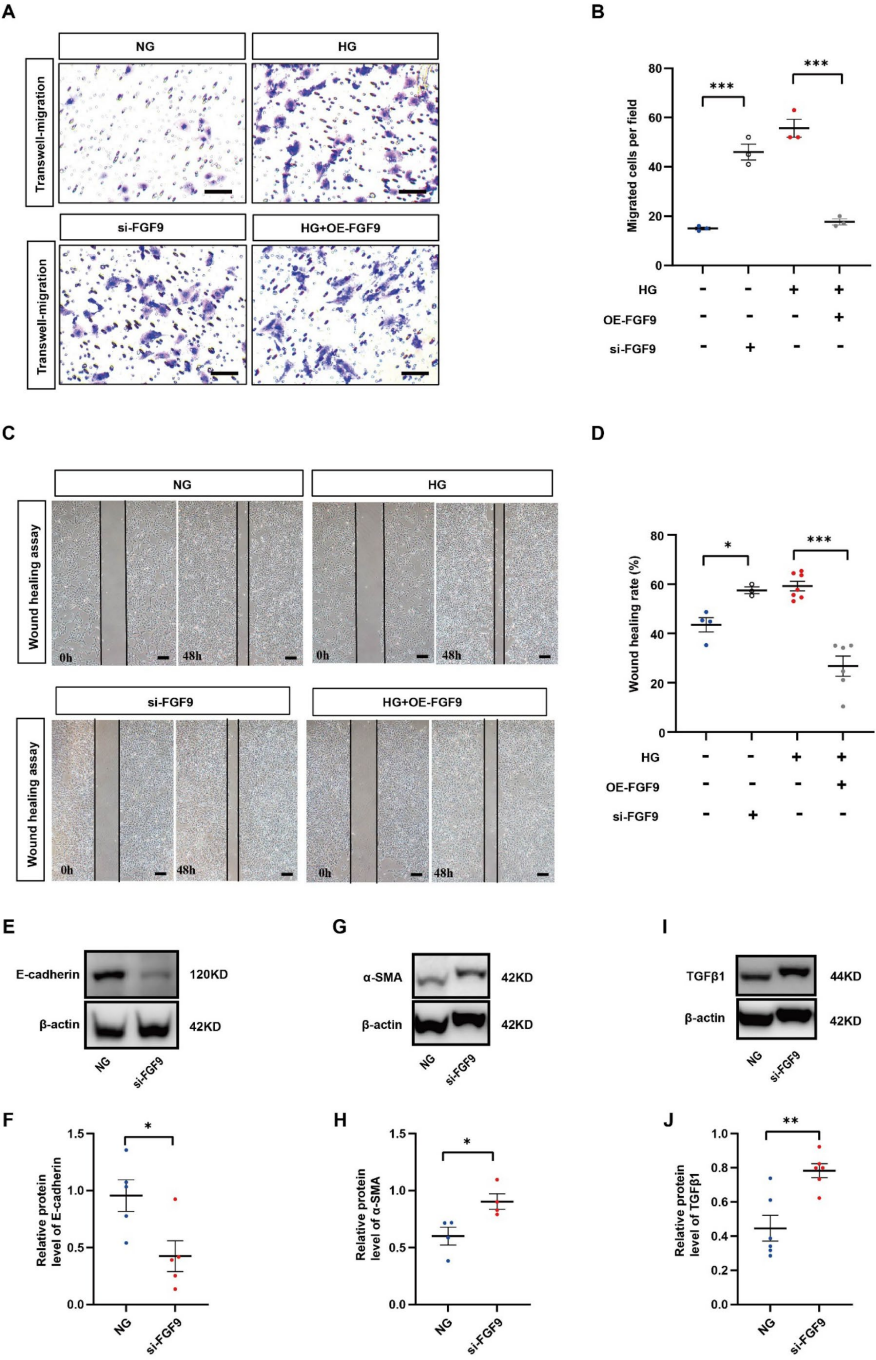
Assessments of cell invasion and migration in HK2 cells. (A, B) Representative images (A) and bar chart (B) of transwell migration assays of HK2 cells with negative control, High glucose (HG) and HG + FGF9‐overexpression vectors after 48 h of incubation. (C, D) Representative images (C) and bar chart (D) of wound healing assays of HK2 cells at 48 h from the above four groups. (E–J) Western blot showing the E‐cadherin (E–F), α‐SMA (G–H) and TGF‐β1 (I–J) protein expression levels in HK2 cells of NG and si‐FGF9. Scale bars = 100 μm in A. *p < 0.05, **p < 0.01, ***p < 0.001.

To further substantiate FGF9's role in the EMT of renal tubular epithelial cells, we manipulated its expression in both 50 mM mannitol (NG) and high glucose (HG) microenvironments. This was achieved using FGF9‐specific siRNA to suppress its expression and FGF9‐overexpressing adenovirus vectors (HG + OE‐FGF9) to increase its expression in human kidney proximal tubular cells (HK2). Our results demonstrated that FGF9 suppression and HG exposure enhanced cell invasion (Figure 5A,B) and migration ability (Figure 5C,D) compared to the NG group, while the overexpression of FGF9 reversed the invasion and migration of HK2 cells in the HG microenvironment when compared to the HG group. Similarly, we investigated the EMT process in HK2 cells. Western blot results indicated an increase in α‐SMA and TGF‐β1 levels, while E‐cadherin exhibited a decrease in the si‐FGF9 group (Figure 5E–J) (Table S9). These findings collectively suggest the occurrence of fibrosis and EMT in the si‐FGF9 group.

### 3.5 Reduced FGF9 Leads to the Upregulation of EMT‐Related Genes Under NG or HG Microenvironment

**FIGURE 6:**
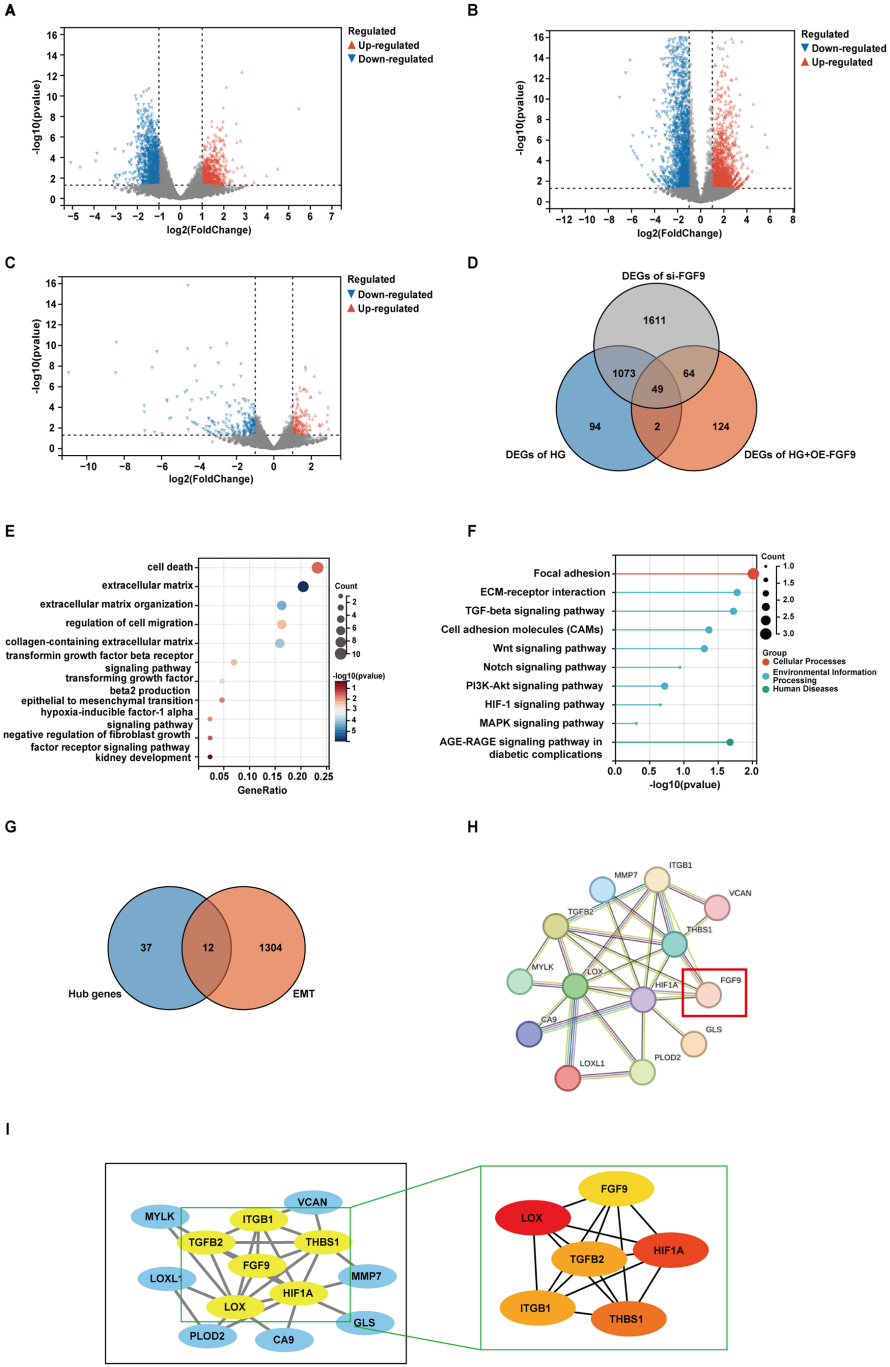
RNA sequencing analysis on renal fibrosis‐related differentially expressed genes in HK2 cells. (A–D) The volcano plot of DEGs with consistency from HG versus NG (A), si‐FGF9 versus NG (B) and HG + OE‐FGF9 versus HG (C). Venn diagram of the DEGs of three combinations (D). (E, F) GO annotation and KEGG pathway enrichment analysis. (G) Intersection of differentially expression genes in HK2 cell of FGF9 knock down and EMT‐related genes. (H) The protein interaction network of the potential relationship between FGF9 and DN‐EMT‐related core targets. (I) FGF9 and topological analysis of DN‐EMT related genes.

To further investigate why the HG suppresses FGF9 and then affects the EMT of renal tubular epithelial cells, we employed RNA sequencing to identify the genes regulated by FGF9. We performed differential analysis between the above four groups: HG versus NG (Figure 6A), si‐FGF9 versus NG (Figure 6B) and HG + OE‐FGF9 versus HG (Figure 6C). The genes that satisfy both |log2Fold Change| ≥ 1 and *p* value < 0.01 with biological significance were selected as differentially expressed genes. Afterwards, the three overlapping differential genes were screened again, thus further screening the downstream target genes after FGF9 intervention. As shown in (Figure 6D), 49 common target genes could be screened.

GO and KEGG analyses of the 49 target genes showed that the target genes were mainly involved in biological processes such as kidney development, cell migration, extracellular matrix, etc. (Figure 6E). KEGG pathway enrichment was mainly enriched in the TGFβ signalling pathway, ECM‐receptor interaction and cell adhesion molecules (Figure 6F). To further understand the relationship between FGF9‐related target genes and EMT, a total of 1316 were identified in the EMT gene database (http://dbemt.bioinfo‐minzhao.org/download.cgi) and the molecular characterisation database v7.1 (http://www.broadinstitute.org/gsea/msigdb/index.jsp) of EMT‐related genes (ERGs). Forty‐nine target genes were localised to ERGs to identify DEGs of FGF9‐associated EMT in DN, resulting in 12 ERGs (Figure 6G).

Subsequently, FGF9 and 12 ERGs above were imported into the String database to generate a PPI network (Figure 6H). Core PPI network modules were obtained using MCODE. The results showed that FGF9 was in the core module, suggesting that FGF9 might be related to these genes. We speculated that the five genes in the core panel, LOX, HIF1α, THBS1, TGFβ2, ITGβ1, might be involved in the diabetes‐induced downregulation of FGF9 to promote the development of renal EMT (Figure 6I). The TGFβ pathway is now recognised as the most important signalling pathway in the transdifferentiation of renal tubular epithelial cells. Figure 6I shows that FGF9 may be associated with TGFβ2. Therefore, we speculate that FGF9 plays an important role in promoting the development of diabetic renal interstitial fibrosis and that FGF9 promotes diabetic tubular transdifferentiation through the FGF9‐TGFβ signalling pathway.

**FIGURE 7:**
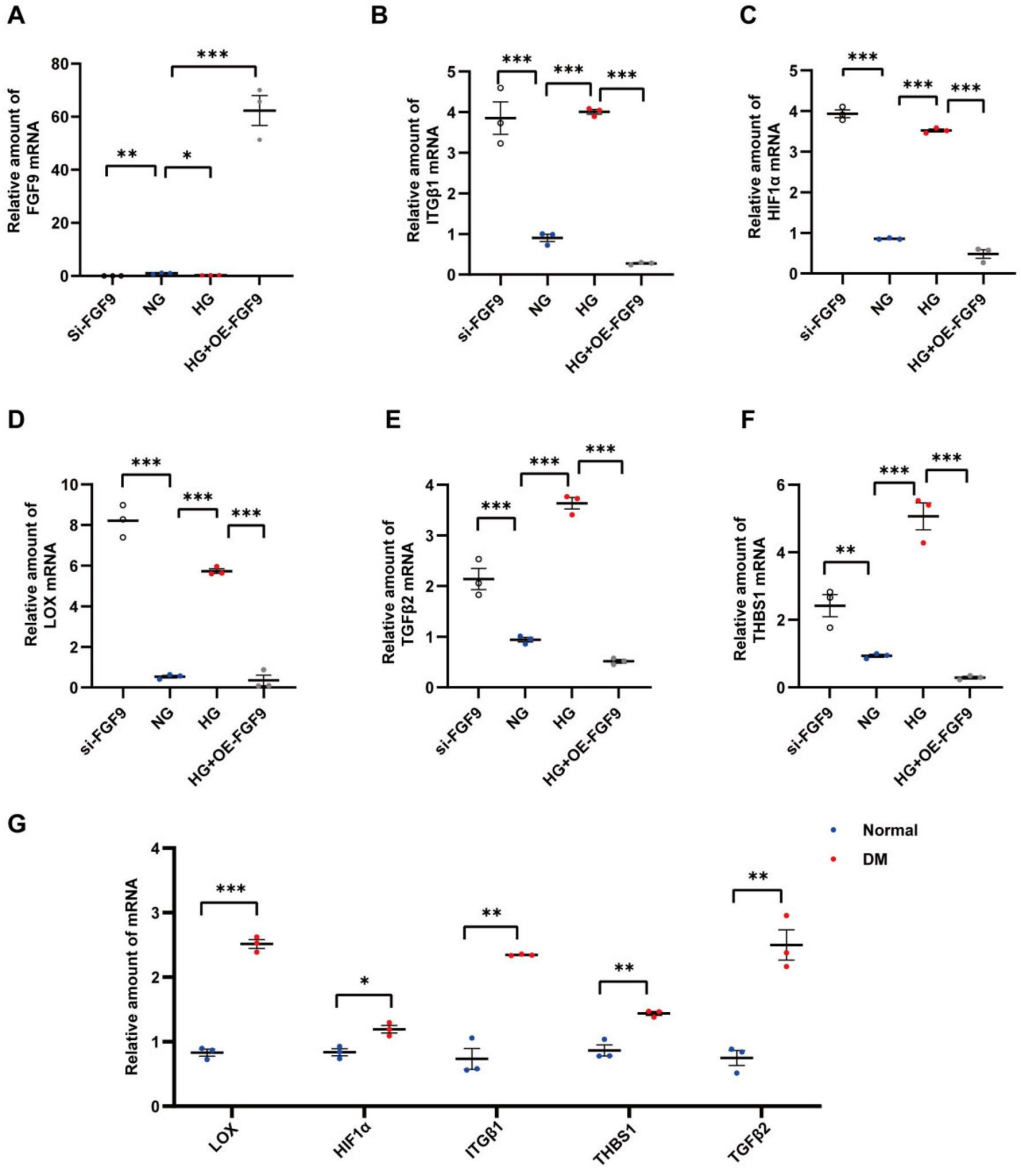
RT‐qPCR data show the relative expressions of EMT related genes at mRNA level in HK2 cell and kidney tissues of the diabetic mouse model. (A–F) mRNA levels of FGF9, ITGβ1, LOX, TGFβ2, THBS1 and HIF1α were assessed in four groups (NG, si‐FGF9, HG and HG + OE‐FGF9) of HK2 cells. (G) mRNA levels of LOX, HIF1α, THBS1, TGFβ2 and ITGβ1were assessed in kidney tissues from Control and diabetic (DM) mice.

In order to verify whether LOX, HIF1α, THBS1, TGFβ2, ITGβ1 would be altered accordingly under the condition of high glucose and FGF9 alteration, we measured the expression of the six genes mentioned above in four groups of HK2 cells: NG, HG, si‐FGF9 and HG + OE‐FGF9. The mRNA levels of the above genes were elevated in the HG and si‐FGF9 groups, while they were decreased in the HG + OE‐FGF9 group. It was further verified that high glucose led to the down‐regulation of FGF9, which further led to the elevation of LOX, HIF1α, THBS1, TGFβ2 and ITGβ1, thereby promoting the development of renal EMT (Figure 7A–F) (Table S10). These data were also validated in kidney tissue of diabetic mice (Figure 7G) (Table S11). Furthermore, we have also confirmed that LOX is upregulated in the si‐FGF9 group at the protein level (Figure S1).

## 4 Discussion

In this study, we conducted a screening of DEGs in DN patient samples using the updated GEO database. The DEGs were subjected to analysis through GO annotation and KEGG pathway enrichment. The results indicated the involvement of cell migration, along with related cell components and molecular functions, in DN pathogenesis. The identification of hub genes was performed through PPI analysis, Cytoscape and the ‘UpSetR’ package. Ultimately, FGF9 emerged as a key candidate based on bioinformatics methodologies. While some previous studies have analysed hub genes associated with DN and suggested that FGF9 might be one of them [39], our study integrated data from multiple RNA sequencing databases, allowing us to focus more specifically on the role of FGF9.

FGF9, a key player in various pathophysiological processes including platelet production [40], bone development, nervous system development [41], kidney development [42] and tumorigenesis [43]. However, its mechanisms in DN remain elusive. Previous studies have hinted at the down‐regulation of FGF1 and FGF9, indicating a weakened antioxidant capacity in DN, rendering the kidney susceptible to oxidative stress injury [44]. Therefore, our first objective was to unravel the expression pattern of FGF9 in kidney tissues of both control and DN patients. In our experiments, we first verified that FGF9 was decreased in both serum and kidney tissues of DN patients. FGF9 is primarily secreted by a variety of cell types, including fibroblasts, chondrocytes, osteoblasts, neuronal cells and other cell types. These cells can produce and release FGF9 in different tissues and organs, and we speculate that the reduced secretion of FGF9 from kidney cells and the reduced release into the circulation in the presence of high glucose may be roughly responsible for further renal fibrosis. At the same time, FGF9 in serum was negatively correlated with blood creatinine and positively correlated with estimated glomerular filtration rate, which provides the possibility of assessing the disease progression and severity by the expression level of FGF9 in serum in the future.

The FGF9 subfamily, consisting of FGF9, FGF16 and FGF20, shares a substantial amino acid sequence similarity of 62%–73% [45, 46]. This subfamily exhibits a high degree of sequence homology between humans and mice [47, 48]. The expression patterns of FGF9 in both humans and mice corroborate RNA sequence data, reinforcing the notion that FGF9 plays a significant role in DN. Moreover, FGF9 predominantly activates FGFR3c > 2c > 1c, 3b ≫ 4 [17], and given the high expression of FGFR2 and FGFR3 in renal tubules, we propose that the suppression of FGF9 might play a pivotal role in renal tubular epithelial cells during DN through FGFR2 and FGFR3.

While albuminuria has traditionally been associated with glomerular damage induced by diabetes, it is increasingly recognised that the renal tubule interstitium also plays a crucial role in the pathogenesis of DN [49], with the EMT process being a key contributor. FGF9 has been reported to mediate EMT in tumour cells. For instance, FGF9 activates FAK, AKT and ERK signalling pathways to induce EMT, thereby promoting lung cancer tumorigenesis and liver metastasis [50]. Additionally, downregulation of miRNA‐214 in gastric cancer results in increased FGF9 expression, thereby promoting the EMT process in gastric cancer cells, ultimately enhancing their migration and invasion abilities in vitro [51]. However, FGF9 exhibits varied responses to EMT in different cells, such as suppressing metastasis in renca cell‐derived renal tumours [52]. In our study, through manipulated FGF9 expression, we confirmed FGF9's involvement in the EMT of renal tubular epithelial cells using Transwell and Wound Healing assays. Specifically, low FGF9 expression in HK2 cells mimicked a high glucose‐like experimental phenotype, leading to increased cell migration and invasion abilities. Conversely, FGF9 overexpression in a high glucose environment inhibited cell migration and invasion. These results underscore the significant role of FGF9 in renal tubular EMT and its potential as a future target for the prevention and treatment of DN.

To delve deeper into the mechanisms underlying the impact of FGF9 suppression or overexpression on EMT, we conducted RNA sequencing analysis in HK2 cells. The results suggest that LOX, HIF1α, THBS1, TGFβ2, and ITGβ1 may be involved in the diabetes‐induced downregulation of FGF9, thus promoting the development of renal EMT. The regulation of HIF‐1 in the diabetic kidney is cell type‐dependent. In tethered cells, high glucose levels increase the expression of HIF‐1α and its target gene ADAM17, thereby accelerating renal fibrosis [53, 54]. ITGβ1 serves as a crucial ECM receptor and is the most abundantly expressed β‐integrin subunit in the kidney [55]. It plays a pivotal role in the regulation of renal structure and function [56]. Recent studies have shown that inhibiting ITGβ1 expression counteracts the TGF‐β1‐induced EMT process in renal tubular epithelial cells and obstructs renal fibrosis in unilateral ureteral obstruction (UUO) mouse models [57]. Serum LOX has been proposed as a potential diagnostic biomarker for renal fibrosis [58]. Upregulation of LOX has been linked to ECM cross‐linking and the progression of renal fibrosis in ischemia–reperfusion injury (IRI) mouse kidneys. Targeting LOX with its inhibitor BAPN significantly reduces IRI‐induced ECM over‐cross‐linking by inhibiting its expression and activity, thereby mitigating renal fibrosis [59]. Previous studies have demonstrated that THBS1 binds to CD47 to promote acute kidney injury (AKI), and blocking THBS1 signalling by CD47 alleviates renal interstitial fibrosis [60]. TGFβ is a key driver of fibrosis. Systemic delivery of CAT‐152, a neutralising anti‐TGF‐β2 antibody, has been shown to reduce the incidence of pathogenic fibrosis in the kidney during the acute phase of DN [61].

In conclusion, our study has revealed a decrease in FGF9 expression within renal tubular cells during DN, as evidenced by comprehensive analyses spanning bioinformatics, clinical samples, animal models and cellular experiments. Moreover, we have established that reduced FGF9 expression primarily facilitates the EMT via FGFR2–3. Our study suggests that FGF9 could be a candidate biomarker and therapeutic target for DN in the future.

## 5 Limitations

To ascertain the viability of FGF9 as a biomarker for DN, it is imperative to conduct future prospective studies with larger sample sizes. Moreover, validating the therapeutic potential of FGF9 for DN could be achieved through targeted overexpression in renal tubules using diabetic mouse models. Furthermore, it is worth exploring whether FGF9 plays a role in other cell types, such as immune cells, which necessitates further investigation.

## Author Contributions

**Wen‐qing Chen:** data curation (equal), formal analysis (equal), investigation (equal), methodology (equal), writing – original draft (equal). **Chengyang Sun:** data curation (equal), formal analysis (equal), investigation (equal), methodology (equal), writing – original draft (equal). **Xiaotan Zhang:** data curation (equal), formal analysis (equal), investigation (equal), methodology (equal), writing – review and editing (equal). **Xunjia Ye:** data curation (equal), formal analysis (equal), investigation (equal), methodology (equal), writing – review and editing (equal). **Yuzhen Liu:** methodology (equal). **Hui‐di Wang:** investigation (supporting), methodology (supporting). **Lichao Liu:** investigation (supporting), methodology (supporting). **Danrui Li:** investigation (supporting), visualization (supporting). **Jingyun Wang:** investigation (supporting), visualization (supporting). **Meiting Shi:** methodology (equal). **Fang Yang:** supervision (supporting), validation (supporting). **Christoph Reichetzeder:** supervision (equal), validation (equal). **Berthold Hocher:** validation (equal), visualization (equal). **Xuesong Yang:** validation (supporting), visualization (supporting). **Baozhang Guan:** funding acquisition (equal), project administration (equal), supervision (equal). **Guang Wang:** funding acquisition (equal), project administration (equal), resources (equal), supervision (equal).

## Ethics Statement

This study was approved by the Ethics Committee of Overseas Hospital, Jinan University, China (approval number: KY‐2023‐222) and conducted in accordance with the Declaration of Helsinki.

## Consent

The authors have nothing to report.

## Conflicts of Interest

The authors declare no conflicts of interest.

## Supporting information


**Figure S1:** FGF9 expressions in human kidney tissues. (A) FGF9 immunohistochemical staining on the cross‐sections of human kidney tissues from healthy kidney from the donors who died from car accident (CON) (A) and damaged regions of the patients with DN (B). The panels shows FGF9 expression in renal glomerulus and tubule respectively. (B) Bar chart showing the comparison of mean optical density values of FGF9 immunohistochemical staining between CON and DN groups. Scale bar = 50 μm in A. ***p < 0.01.

**Data S1:** jcmm70856‐sup‐0002‐Supinfo.pdf.

## Data Availability

RNA sequencing (RNA‐seq) data were deposited in the National Center for Biotechnology Information GenBank (BioProject ID: GSE265918). The remaining data are available within the article and Supporting Information.
