# Supplementary material for: Reduced FGF9 Leads to Kidney Injury Through Regulating Renal Tubular Epithelial Cell EMT in Diabetes
Source: J Cell Mol Med. 2025 Sep 22;29(18):e70856. doi: 10.1111/jcmm.70856 (PMC12451398; doi:10.1111/jcmm.70856)
Supplement: Supplementary file 1 — Figure S1: FGF9 expressions in human kidney tissues. (A) FGF9 immunohistochemical staining on the cross‐sections of human kidney tissues from healthy kidney from the donors who died from car accident (CON) (A) and damaged regions of the patients with DN (B). The panels shows FGF9 expression in renal glomerulus and tubule respectively. (B) Bar chart showing the comparison of mean optical density values of FGF9 immunohistochemical staining between CON and DN groups. Scale bar = 50 μm in A. ***p < 0.01. [file JCMM-29-e70856-s002.docx]

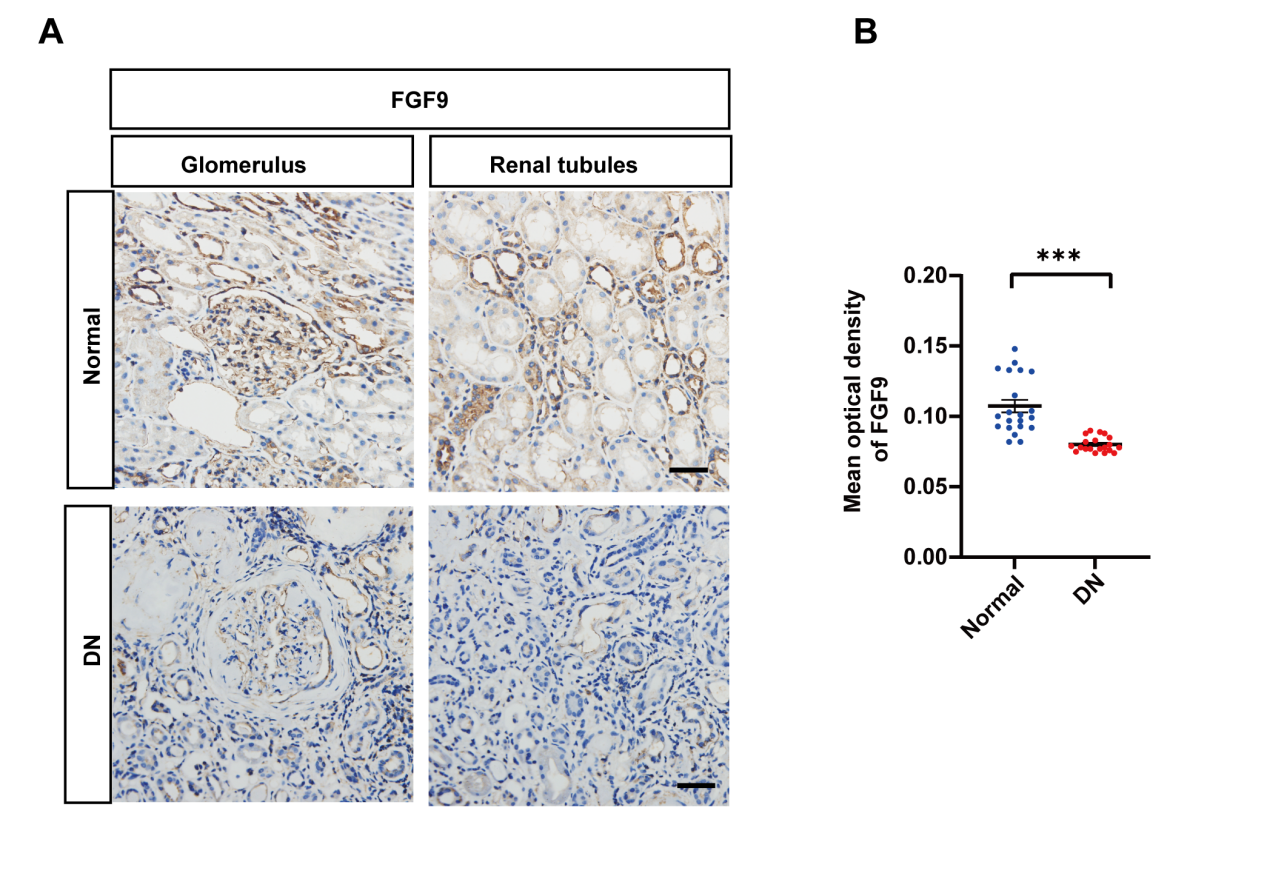


***Fig. S1. FGF9 expressions in human kidney tissues.***

**A:** FGF9 immunohistochemical staining on the cross-sections of human kidney tissues from healthy kidney from the donors who died from car accident (CON) (A) and damaged regions of the patients with DN (B). The pannels shows FGF9 expression in renal glomerulus and tubule respectively. **B:** Bar chart showing the comparison of mean optical density values of FGF9 immunohistochemical staining between CON and DN groups. Scale bar = 50 μm in A. ****P* < 0.01.
