# Supplementary material for: Reduced FGF9 Leads to Kidney Injury Through Regulating Renal Tubular Epithelial Cell EMT in Diabetes
Source: J Cell Mol Med. 2025 Sep 22;29(18):e70856. doi: 10.1111/jcmm.70856 (PMC12451398; doi:10.1111/jcmm.70856)
Supplement: Supplementary file 2 — Data S1: jcmm70856‐sup‐0002‐Supinfo.pdf. [file JCMM-29-e70856-s001.pdf]

Supplementary Tables

Supplementary Table 1. Primer sets used for RT-qPCR studies

Gene	Forward primer	Reverse primer
18sRNA(m)	ACACGGACAGGATTGACAGA	GGACATCTAAGGGCATCACAG
FGF9(m)	GGGGAGCTGTATGGATCAGA	GTGAATTTCTGGTGCCGTTT
18sRNA(h)	TCAACTTTCGATGGTAGTCGCCGT	TCCTTGGATGTGGTAGCCGTTTCT
FGF9(h)	CAGGCGGAGGCAGCTATAC	CCTGGTTCCCTGGATAGTACC
LOX(h)	CCCTCCTGCTTCCTTTTCACA	GAATGTCACAGCGCACAACA
HIF1α(h)	TCCAAGAAGCCCTAACGTGT	TTTCGCTTTCTCTGAGCATTCTG
THBS1 (h)	GCTGGAAATGTGGTGCTTGTCC	CTCCATTGTGGTTGAAGCAGGC
ITGβ1(h)	TGGGAAACTTGGTGGC	CTCCTTGTAAACAGGCTG
TGFβ2(h)	AAGAAGCGTGCTTTGGATGCGG	ATGCTCCAGCACAGAAGTTGGC
ITGAV(h)	GACAGTCCTGCCGAGTA	CTGGGTGGTGTTTGCT

Supplementary Table2. Antibodies for Immunohistochemistry staining

Antibody	Concentration	Article Number	Company	Country
FGF9	1:200	sc-398730	Santa Cruz	CA
FGF9	1:200	DF-9532	Affinity Biosciences	China
α-SMA	1:300	ab5694	Abcam	UK
FGFR1	1:100	bs-0230R	Bioss	China
FGFR2	1:100	bs-0675R	Bioss	China
FGFR3	1:100	bs-1301R	Bioss	China
FGFR4	1:100	bs-0676R	Bioss	China

Supplementary Table 3. Antibodies for Western blotting

Antibody	Concentration	Article Number	Company	Country
FGF9	1:1000	sc-398730	Santa Cruz	CA
α-SMA	1:1000	ab5694	Abcam	UK
E-cadherin	1:3000	ab231303	Abcam	UK
TGFβ1	1:1000	21898-1-AP	Proteintech	USA
β-actin	1:10000	HRP-60008	Proteintech	USA

Supplementary Table 4. The statistical description and statistical results in Figure 1

	Normal	DN	Pvalue
Fig.1K			
FGF9	8.33±0.63(n=14)	7.80±0.30(n=7)	0.017

Supplementary Table 5. The statistical description and statistical results in Figure S1

	Normal	DN	Pvalue
Fig.S1B			
FGF9	0.11±0.02(n=21)	0.08±0.005(n=20)	< 0.001

Supplementary Table6. The statistical description and statistical results in Figure 2

	Contral	DM	Pvalue
Fig.2B(n=10 per group)			
10 weeks	5.68±0.61	19.12±0.74	<0.001
16 weeks	6.21±0.51	23.72±0.68	<0.001
22 weeks	6.35±1.36	27.00±0.92	<0.001
Fig.2C	7.42±2.64(n=7)	30.06±3.91(n=7)	<0.001
Fig.2D	6.13±2.13(n=7)	73.00±25.87(n=7)	<0.001
Fig.2F	1.84±0.55(n=20)	5.76±2.83(n=20)	<0.001
Fig.2G	0.01±0.01(n=12)	2.80±2.12(n=15)	<0.001
Fig.2I	0.06±0.01(n=29)	0.04±0.01(n=54)	<0.001
Fig.2J	1.0±0.28(n=9)	0.42±0.43(n=9)	0.004
Fig.2L	1.66±1.00(n=21)	0.71±0.21(n=18)	<0.001

Supplementary Table7. The statistical description and statistical results in Figure 3

	Contral	DM	Pvalue
Fig.3B	0.07±0.02(n=15)	0.15±0.02(n=15)	<0.001
Fig.3C	0.25±0.05(n=15)	0.17±0.02(n=15)	<0.001
Fig.3D	0.26±0.04(n=15)	0.13±0.01(n=15)	<0.001
Fig.3E	0.23±0.44(n=15)	0.21±0.01(n=15)	0.178

Supplementary Table8. The statistical description and statistical results in Figure 4

	Contral	DM	Pvalue
Fig.4B	0.34±0.29(n=7)	0.88±0.39(n=9)	0.009
Fig.4C	0.83±0.24(n=9)	1.28±0.36(n=9)	0.007
Fig.4D	1.07±0.38(n=9)	0.65±0.06(n=9)	0.01
Fig.4F	0.02±0.01(n=33)	0.04±0.01(n=32)	<0.001

Supplementary Table9. The statistical description and statistical results in Figure 5

	NG	Si-FGF9	HG	HG+OE-FGF9	Pvalue
Fig.5B	15.0±1.0(n=3)	46.0±5.57（n=3）			<0.001
			55.67±6.35(n=3)	17.67±2.08(n=3)	<0.001
Fig.5D	43.52±5.81(n=4)	57.51±2.34(n=3)			0.018
			59.22±5.14(n=7)	26.73±9.98(n=6)	<0.001
Fig.5F	0.96±0.31(n=5)	0.42±0.30(n=5)			0.025
Fig.5H	0.60±0.16(n=4)	0.90±0.14(n=4)			0.027
Fig.5J	0.45±0.18(n=6)	0.78±0.10(n=6)			0.003

Supplementary Table10. The statistical description and statistical results in Figure 7

	Si-FGF9	NG	HG	HG+OE-FGF9	Pvalue
Fig.7A		1.0±0.32(n=3)	0.27±0.02(n=3)		0.017
	0.03±0.19(n=3)	1.0±0.32(n=3)			0.06
		1.0±0.32(n=3)		62.33±9.81(n=3)	<0.001
Fig.7B		0.90±0.16(n=3)	4.01±0.10(n=3)		<0.001
	3.85±0.69(n=3)	0.90±0.16(n=3)			0.002
			4.01±0.10(n=3)	0.27±0.03(n=3)	<0.001
Fig.7C		0.86±0.02(n=3)	3.52±0.06(n=3)		<0.001
	3.93±0.16(n=3)	0.86±0.02(n=3)			<0.001
			3.52±0.06(n=3)	0.48±0.18(n=3)	<0.001
Fig.7D		0.54±0.12(n=3)	5.73±0.19(n=3)		<0.001
	8.22±0.80(n=3)	0.54±0.12(n=3)			<0.001
			5.73±0.19(n=3)	0.35±0.46(n=3)	<0.001
Fig.7E		0.94±0.08(n=3)	3.63±0.20(n=3)		<0.001
	2.14±0.36(n=3)	0.94±0.08(n=3)			0.005
			3.63±0.20(n=3)	0.51±0.06(n=3)	<0.001
Fig.7F		0.93±0.07(n=3)	5.07±0.69(n=3)		<0.001
	2.42±0.57(n=3)	0.93±0.07(n=3)			0.011
			5.07±0.69(n=3)	0.29±0.06(n=3)	<0.001

Supplementary Table11. The statistical description and statistical results in Figure 7

	Contral	DM	Pvalue
Fig.7G			
LOX	0.83±0.09(n=3)	2.51±0.12(n=3)	< 0.001
HIF1α	0.84±0.1(n=3)	1.19±0.10(n=3)	0.012
ITGβ1	0.73±0.28(n=3)	0.34±0.01(n=3)	0.001
THBS1	0.87±0.15(n=3)	1.44±0.05(n=3)	0.003
TGFβ2	0.75±0.20(n=3)	2.50±0.41(n=3)	0.003

Supplementary Figures

Supplementary Fig.2

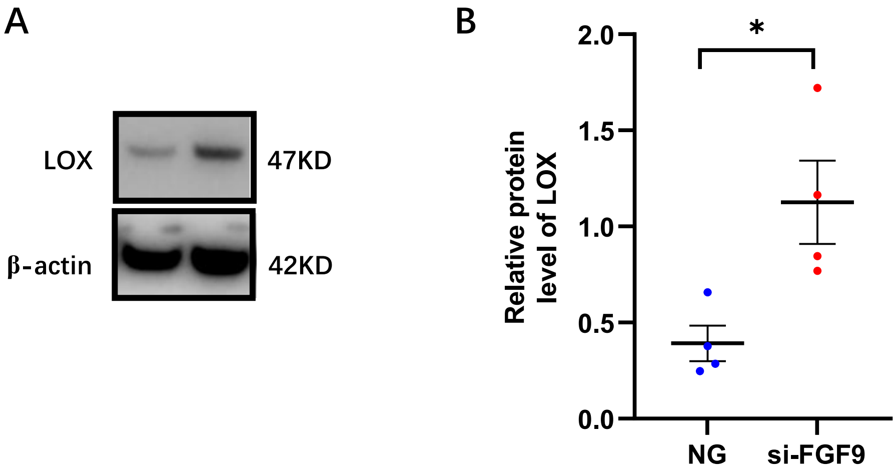

Supplementary Fig.2. LOX protein expression level in HK2 cells of NG and si-FGF9.
